# Human immunodeficiency virus Tat impairs mitochondrial fission in neurons

**DOI:** 10.1038/s41420-017-0013-6

**Published:** 2018-02-02

**Authors:** Summer J. Rozzi, Valeria Avdoshina, Jerel A. Fields, Italo Mocchetti

**Affiliations:** 1 Interdisciplinary Program in Neuroscience, Georgetown University Medical Center, Washington, DC USA; 2 Laboratory of Preclinical Neurobiology, Department of Neuroscience, Georgetown University Medical Center, Washington, DC USA; 3 Department of Psychiatry, University of California San Diego, La Jolla, CA USA

## Abstract

Human immunodeficiency virus-1 (HIV) infection of the central nervous system promotes neuronal injury that culminates in HIV-associated neurocognitive disorders. Viral proteins, including transactivator of transcription (Tat), have emerged as leading candidates to explain HIV-mediated neurotoxicity, though the mechanisms remain unclear. Tat transgenic mice or neurons exposed to Tat, which show neuronal loss, exhibit smaller mitochondria as compared to controls. To provide an experimental clue as to which mechanisms are used by Tat to promote changes in mitochondrial morphology, rat cortical neurons were exposed to Tat (100 nM) for various time points. Within 30 min, Tat caused a significant reduction in mitochondrial membrane potential, a process that is regulated by fusion and fission. To further assess whether Tat changes these processes, fission and fusion proteins dynamin-related protein 1 (Drp1) and mitofusin-2 (Mfn2), respectively, were measured. We found that Drp1 levels increased beginning at 2 h after Tat exposure while Mfn2 remained unchanged. Moreover, increased levels of an active form of Drp1 were found to be present following Tat exposure. Furthermore, Drp1 and calcineurin inhibitors prevented Tat-mediated effects on mitochondria size. These findings indicate that mitochondrial fission is likely the leading factor in Tat-mediated alterations to mitochondrial morphology. This disruption in mitochondria homeostasis may contribute to the instability of the organelle and ultimately neuronal cell death following Tat exposure.

## Introduction

Human immunodeficiency virus type 1 (HIV) causes HIV-associated neurocognitive disorders (HAND) in nearly one-third of individuals^1^. Post-mortem brains from subjects with the most severe form of HAND exhibit neuronal loss accompanied by synaptic simplification, dendritic pruning, loss of spines, degradation of synaptic proteins^2^, and neuronal apoptosis^3,4^. These neurotoxic properties of HIV have been attributed to the combined effect of host cell-derived factors, including cytokines and glutamate, and other neurotoxins produced by activated microglia/macrophages^5^. Moreover, different viral proteins have been shown to directly cause this type of neuronal degeneration, including transactivator of transcription (Tat)^6^, a 101-amino-acid protein that regulates transcription from the HIV promoter^7^. Tat is actively secreted from infected astrocytes, microglia, and macrophages, and can be rapidly internalized by a variety of cell types, including neurons^8^. This internalization has been reported to promote trimming of neurites, mitochondrial dysfunction, and cell death in neurons^9^.

Loss of mitochondrial membrane potential^10,12^ as well as morphologic and functional changes in mitochondria^12,13^ is seen in neurons exposed to Tat. In addition, Tat exposure to rat primary neurons leads to rapid release of reactive oxygen species (ROS) and an increase in 3-(4,5-dimethylthiazol-2-yl)-2,5-diphenyltetrazolium bromide^14^, suggesting impaired mitochondrial activity. This scenario mirrors the mitochondrial irregularities observed in the cortex of patients with HIV encephalitis^15,16^. Efficient mitochondrial function is essential for the health of highly energetic and polarized neurons. The opposite leads to the overproduction of cellular waste products and loss of ATP, both of which can contribute to neuronal cell death^17^. These considerations underscore the important functional relationship between HIV, mitochondria, and neuronal survival. However, to date, few investigations have detailed the mechanisms behind these impairments.

Regulation of mitochondria health is tightly controlled by the dynamic processes of fusion and fission, which in turn, directly affect the size of these organelles and their ability to be trafficked throughout sub-compartments of the neuron^18,19^. Aberrations to any of these processes can contribute to organelle inefficiencies, impair cellular functions, and lead to cell death^20^. Mitochondrial dynamics are processes mediated by GTPases, including dynamin-related protein 1 (Drp1) for fission and mitofusins (Mfn) 1 and 2 for fusion^21,22^. Upon post-translational modification, Drp1 translocates to the mitochondria membrane where it oligomerizes, forms a band around the mitochondria, and promotes fission of the organelle^23^. Acting in an opposite fashion are Mfns, which interact with the outer mitochondrial membrane of two adjacent organelles to induce mitochondrial fission^24^. Mitochondria that accumulate defects in proteins and mitochondrial DNA must be either repaired by fusion with healthy mitochondria or cleared from the cell by selective autophagy^25^. Damaged mitochondria might be transported back to the cell body to be replenished or degraded. Thus, in order to keep energy homeostasis and maintain essential activities, neurons must precisely establish an adequate distribution of mitochondria and also efficiently sustain them in the periphery and clear them away when necessary.

Here we have sought to determine how Tat impairs mitochondrial dynamics in neurons, contributing to cell death. We show that Tat impairs mitochondria membrane potential shortly after exposure and subsequently leads to alterations in mitochondrial size and subcellular localization in a calcineurin-dependent manner.

## Results

### Mitochondria are smaller and fragmented in brains of Tat mice

**Fig. 1:**
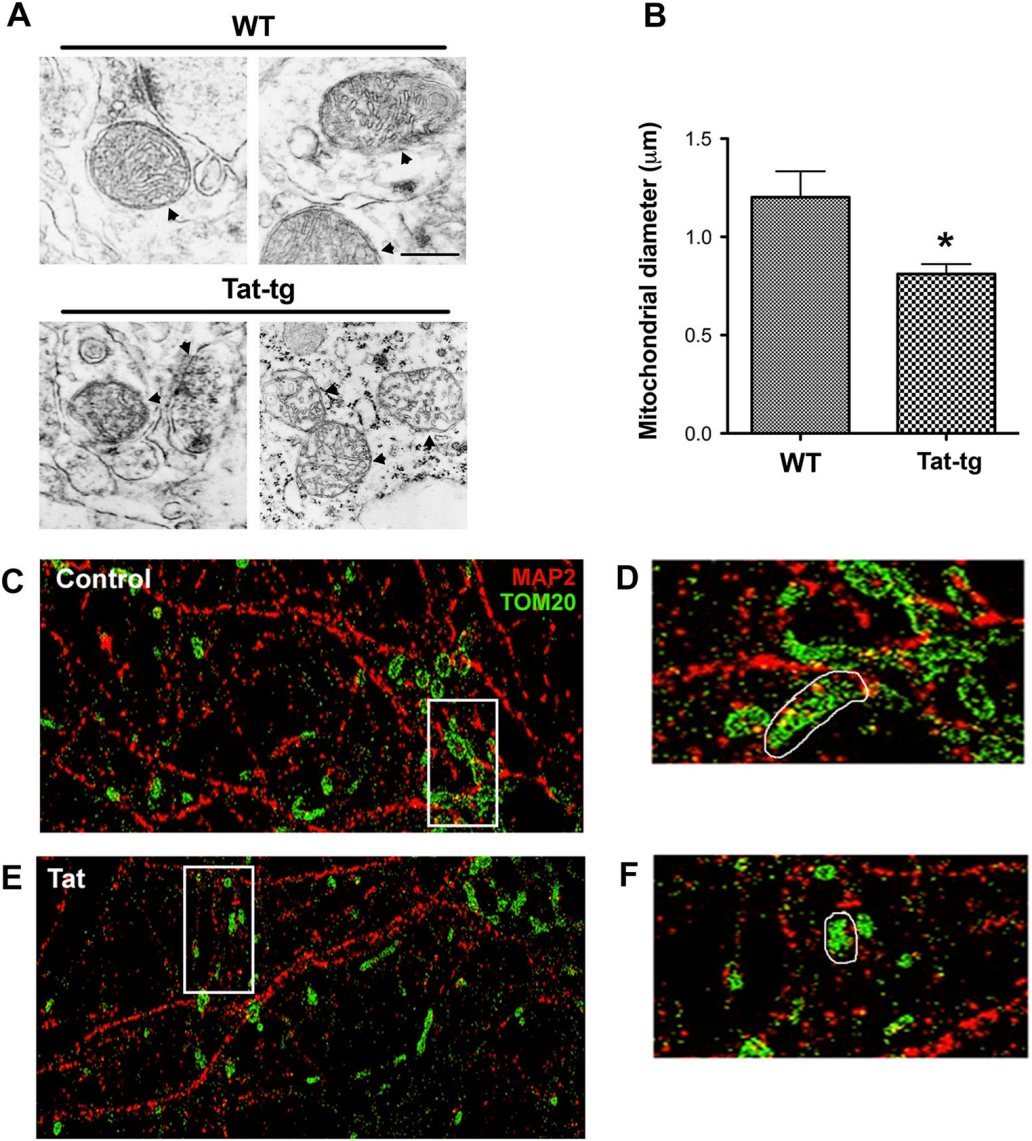
Mitochondrial diameter is decreased in neurons by Tat. **a** WT and Tat-tg mice were treated for 2 weeks with doxycycline. Vibratome sections of their brains were analyzed for mitochondrial morphology by transmission electron microscopy. Arrowheads point at mitochondria of the brains of two separate mice, each group. Scale bar = 500 nm. **b** Average quantification of diameter of neuronal mitochondria in WT and Tat-tg mouse brains. *p < 0.05 by Student’s *t*-test; *n* = 16 (8 WT and 8 Tat-tg mice). **c**–**f** Cortical neurons were fixed with 4% paraformaldehyde + 4% sucrose (pH 7.4) following exposure to control media (**c**) or media containing Tat (100 nM) (**e**) for 4 h. Cells were then stained for MAP2 (red) and TOM20 (green) to label neuronal microtubules and mitochondria, respectively. Images were acquired by STORM as previously described^15^. **d**, **f** Enlargements (×5) of squares in **c** and **e**, respectively

Impaired mitochondrial metabolism^26^ and damaged mitochondria cristae^15,16^ have been observed in HIV-positive subjects. These effects could be due to the toxicity of combined antiretroviral therapy^27,28^ or soluble viral proteins, such as Tat. Mice overexpressing Tat in the brain develop neurodegeneration similar to HIV+ subjects that were diagnosed with HAND^29^. Therefore, we used Tat transgenic (Tat-tg) mice to establish whether Tat alters the morphology of mitochondria. Analyses of mouse brain sections with electron microscopy showed that Tat promotes a significant reduction in mitochondria size when compared to wild-type (WT) littermates (Fig. 1a, b). Indeed, in WT mice, neuronal mitochondrial diameter averaged ~1.2 μm, while in Tat-tg animals neuronal mitochondrial diameter was ~0.8 μm (Fig. 1b).

**Fig. 2:**
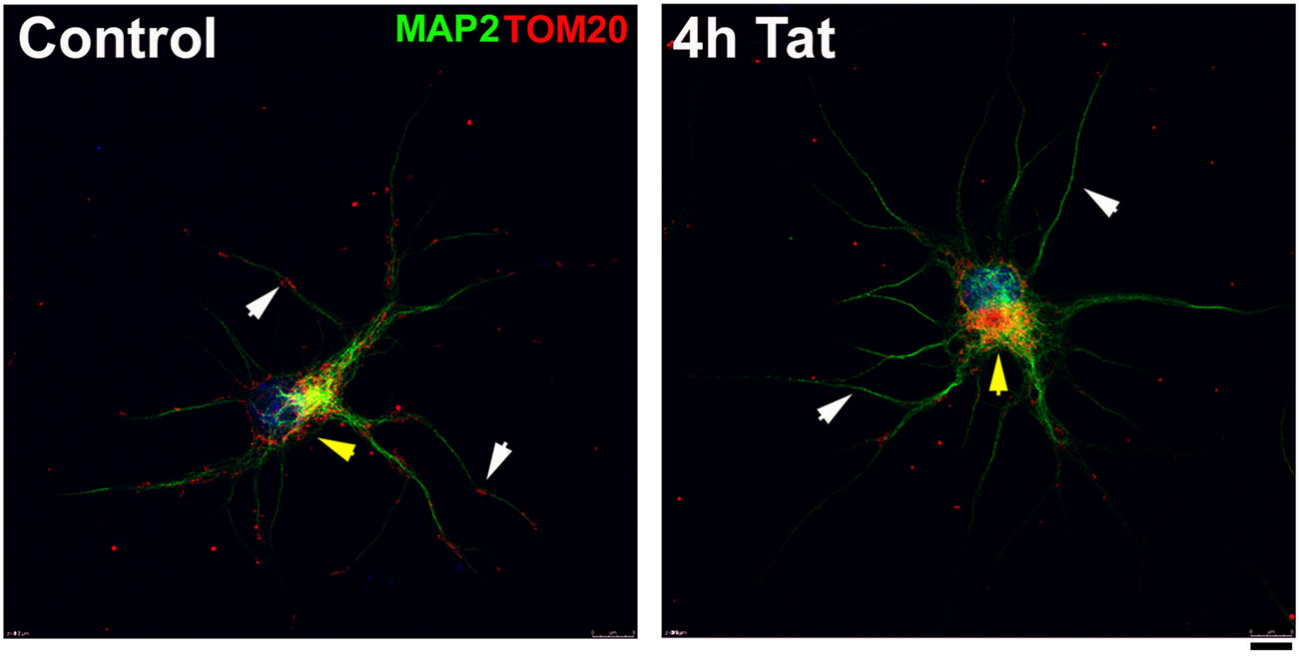
Tat changes the subcellular distribution of mitochondria. Cortical neurons were exposed to boiled Tat (control) or Tat (100 nM) for up to 4 h. Mitochondria were visualized by TOM20 (red) in neurons identified by MAP2 (green) and counterstained with DAPI (blue). All images were acquired at ×40 magnification, Zoom 2. Scale bar = 8 µm. Note that most of mitochondria in control neurons are localized in processes (white arrowheads), those in Tat-treated neurons are localized perinuclearly (yellow arrowhead)

To further confirm the effect of Tat on neuronal mitochondria, stochastic optical reconstruction microscopy (STORM) was used to analyze rat cortical neurons exposed to Tat. Mitochondria were visualized with a TOM20 antibody in MAP2-positive cells (Fig. 1c). Neurons were exposed for up to 4 h to Tat. This is to establish whether changes in mitochondria size precede Tat-mediated neuronal degeneration, which typically occurs in vitro by 24 h^30,31^. In Tat-treated neurons (Fig. 1e–f), we observed smaller mitochondria compared to controls (Fig. 1c, d). Moreover, beginning at 2 h (data not shown) and up to 4 h (Fig. 2), TOM20 immunoreactivity in Tat-treated neurons began to be seen less in the processes and more within the soma, suggesting that Tat also alters subcellular distribution of mitochondria.

### Tat impairs mitochondrial membrane potential

**Fig. 3:**
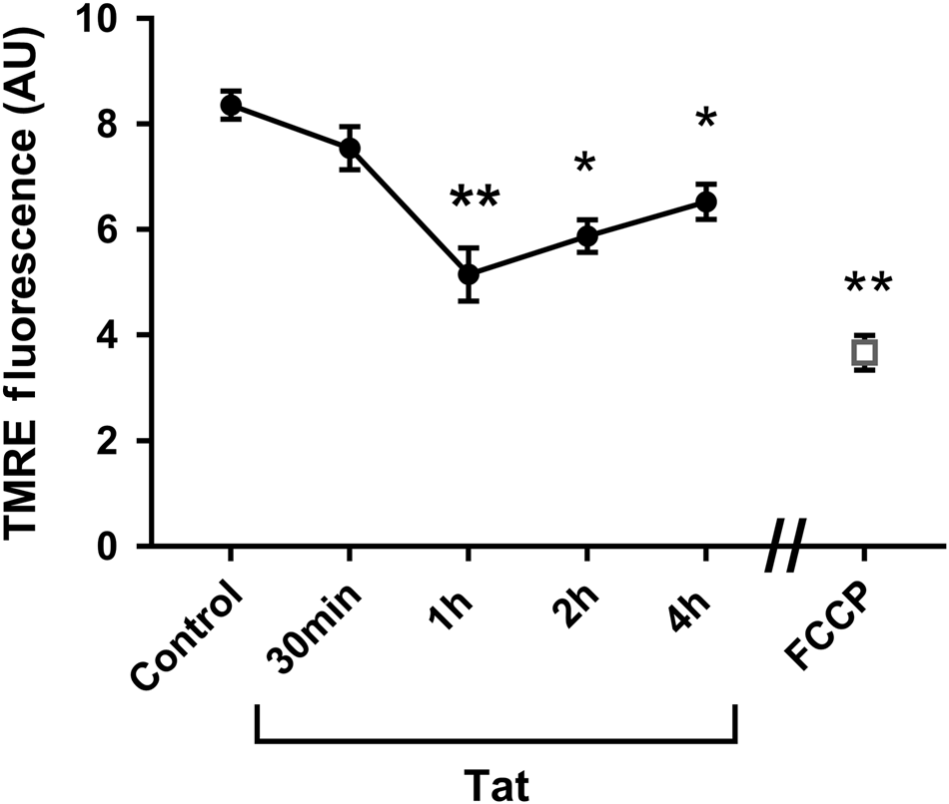
Tat elicits a time-dependent decrease in mitochondrial membrane potential. Cortical neurons were exposed to boiled Tat (control), Tat (100 nM) for the indicated times, or to FCCP (0.5 µM) for 30 min. Cells were then loaded with mitochondrial membrane permeant and potential-dependent dye, TMRE (5 nM), for 30 min (see Materials and Methods). The fluorescent intensity was quantified using ImageJ and expressed in arbitrary units (AU). Data are the mean ± SEM (*n* = 20 coverslips each time point). *p < 0.001, **p < .0001 vs control (ANOVA and Tukey’s test). TMRE tetramethylrhodamine ethyl ester

To examine whether Tat changes the mitochondrial membrane potential, we determined the accumulation of tetramethylrhodamine ethyl ester (TMRE), a permeant dye that is selectively taken up by mitochondria in relation to their membrane potentials. Lower TMRE fluorescence indicates mitochondrial depolarization. Cortical neurons were exposed to Tat for various time points and mitochondria membrane potential was analyzed by TMRE uptake. Carbonilcyanide *p*-triflouromethoxyphenylhydrazone (FCCP), an uncoupler of mitochondria oxidative phosphorylation, was used as a positive control for the assay. By 30 min, Tat-treated neurons exhibited a decreased fluorescent intensity compared to controls neurons (heat-inactivated Tat) imaged under the same conditions (Fig. 3). The decrease in fluorescence intensity was significant by 1 h and up to 4 h (Fig. 3), suggesting the destabilization of the organelles.

### Tat decreases mitochondrial size without altering mitochondrial number

**Fig. 4:**
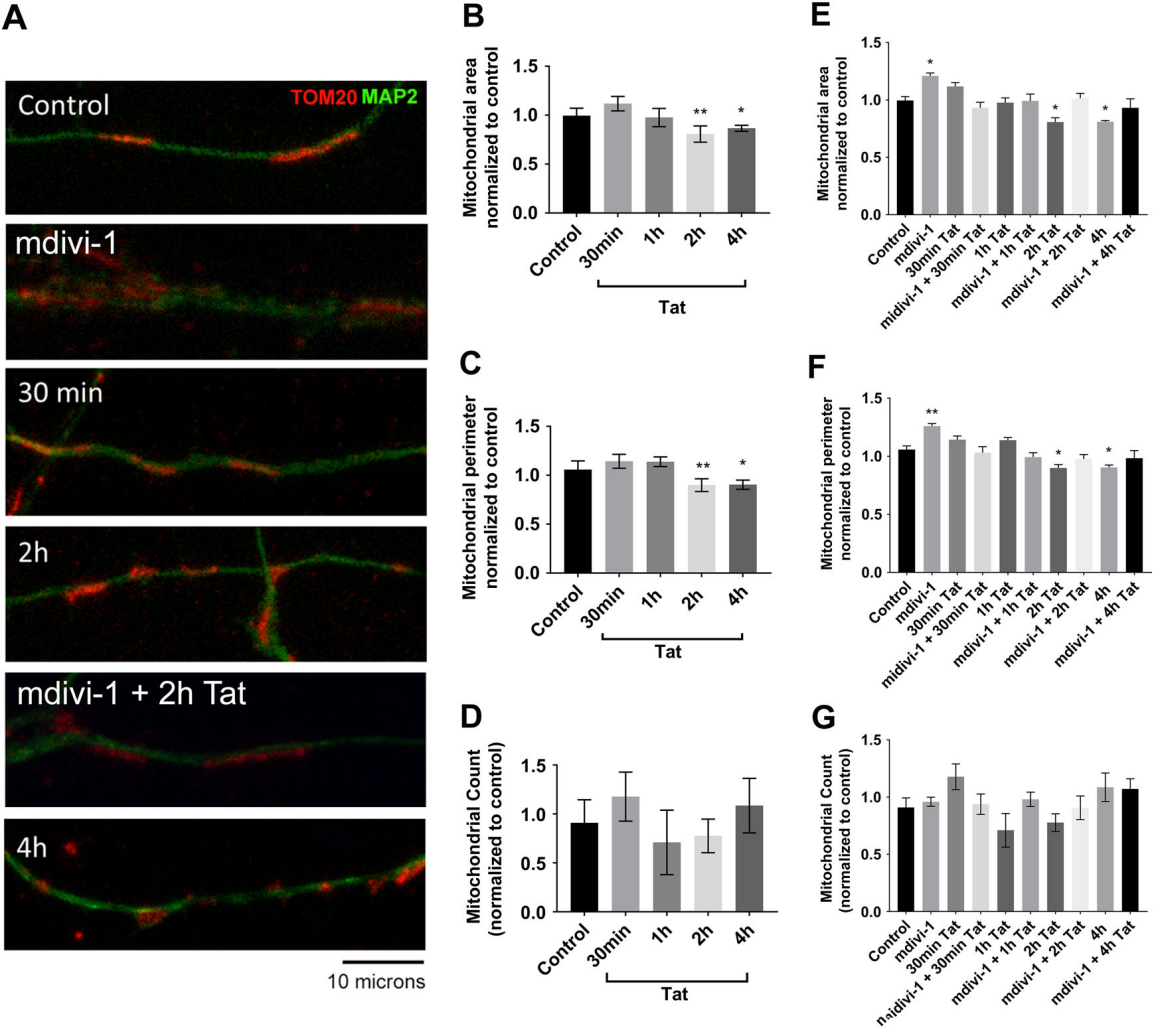
Tat decreases mitochondrial size without altering mitochondrial number. Cortical neurons were exposed to boiled Tat (control) or Tat (100 nM) for the specified time points alone (**b**–**d**) or in combination with mdivi-1 (10 μM) (**e**–**g**). **a** Cells were then fixed and stained for MAP2 (green) and TOM20 (red). Quantification of mitochondrial area (**b**, **e**), perimeter (**c**, **f**), and number (**d**, **g**) was then done as described in Materials and Methods. Data are the mean ± SEM of 20 neurons per treatment, normalized to control. *p < 0.05, **p < 0.01 vs control (ANOVA and Tukey’s test)

Changes in mitochondrial membrane potential can be indicative of alterations of mitochondrial morphology and number. To test for these possibilities, we examine the effect of Tat using cortical neurons. Mitochondria were visualized by TOM20 immunoreactivity (Fig. 4a). Tat elicited a time-dependent decrease in mitochondrial area (Fig. 4b) and perimeter (Fig. 4c), without changing their number (Fig. 4d). To examine whether changes in morphology could be due to fragmentation, neurons were also exposed to mitochondrial division inhibitor 1 (mdivi-1), a cell-permeable inhibitor of mitochondrial division. Mdivi-1 inhibited the ability of Tat to decrease mitochondrial area (Fig. 4e) and perimeter (Fig. 4f) at any time points and had no effect on total mitochondria number (Fig. 4g), suggesting that Tat may increase mitochondrial fragmentation.

### Tat exposure leads to an increase in levels of Drp1

**Fig. 5:**
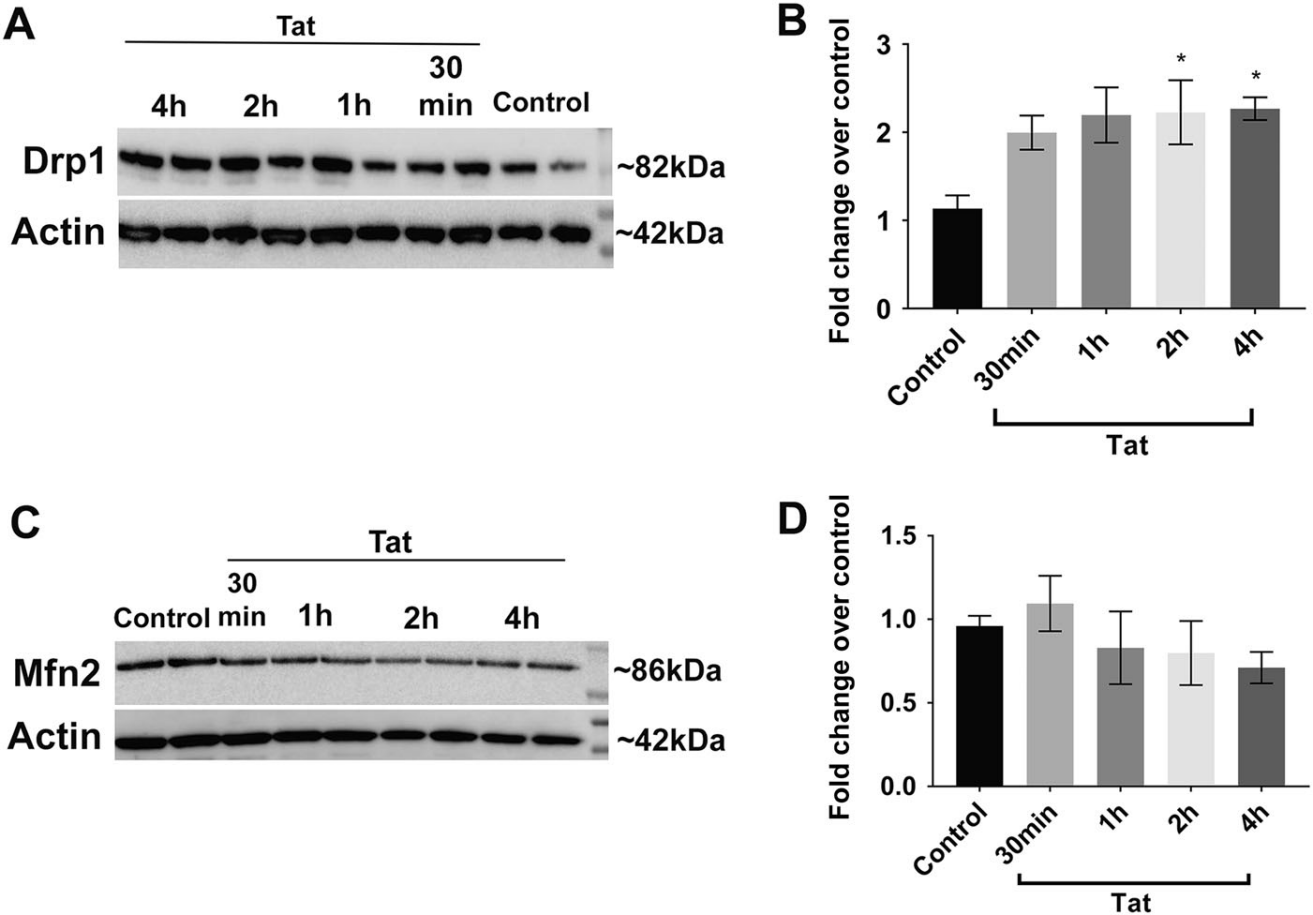
Tat promotes a time-dependent increase in Drp1, but not Mfn2 levels. Drp1 and Mfns protein levels were determined by western blot analysis in control and Tat-treated neurons. **a** Representative western blot analysis of cortical neuronal lysates probed with a Drp1 antibody. Blots were reprobed with beta-actin antibody. **b** Semi-quantification of Drp1 levels was done by densitometric analysis of the 82 kDa immunoreactive band normalized by the beta-actin (42 kDa) immunoreactivity. **c** Representative western blot analysis of cortical neuronal lysates probed with a Mfn2 antibody. **d** Semi-quantification of Mfn2 levels was done by densitometric analysis of the 86 kDa immunoreactive band normalized by beta-actin immunoreacivity. Data are the mean ± SEM of three independent experiments, normalized to control. *p < 0.05 vs control (ANOVA and Tukey’s test)

Mdivi-1 reduces the activation of the regulator of fission Drp1. Fission and fusion are important processes in mitochondrial dynamics because perturbations to either can lead to morphological disturbances of the organelle and neuronal degeneration. Thus, we examined whether Tat changes the levels of the proteins regulating fusion and fission. First, we determined the levels of Drp1 by western blot analysis on lysates collected from neuronal cultures exposed to Tat for 30 min and up to 4 h, and probed with antibodies against Drp1. The antibody detected an immunoreactive band at ~ 82 kDa (Fig. 5a), which is typical for Drp1. Densitometric analysis revealed that Tat significantly increased Drp1 levels compared to control by 2 h (Fig. 5b). The increase persisted for up to 4 h (Fig. 5a, b).

Next, to determine if Tat alters proteins regulating mitochondrial fusion, we analyzed same lysates with an antibody against Mfn2. Unlike the changes we observed following Tat exposure in Drp1 protein levels, Mfn2 levels were not significantly different than control (Fig. 5c, d). Taken together, these results indicate that the increased fragmentation of mitochondria is likely due to an increase in fission activity and not a decrease in fusion.

### Tat decreases the phosphorylation of Drp1

**Fig. 6:**
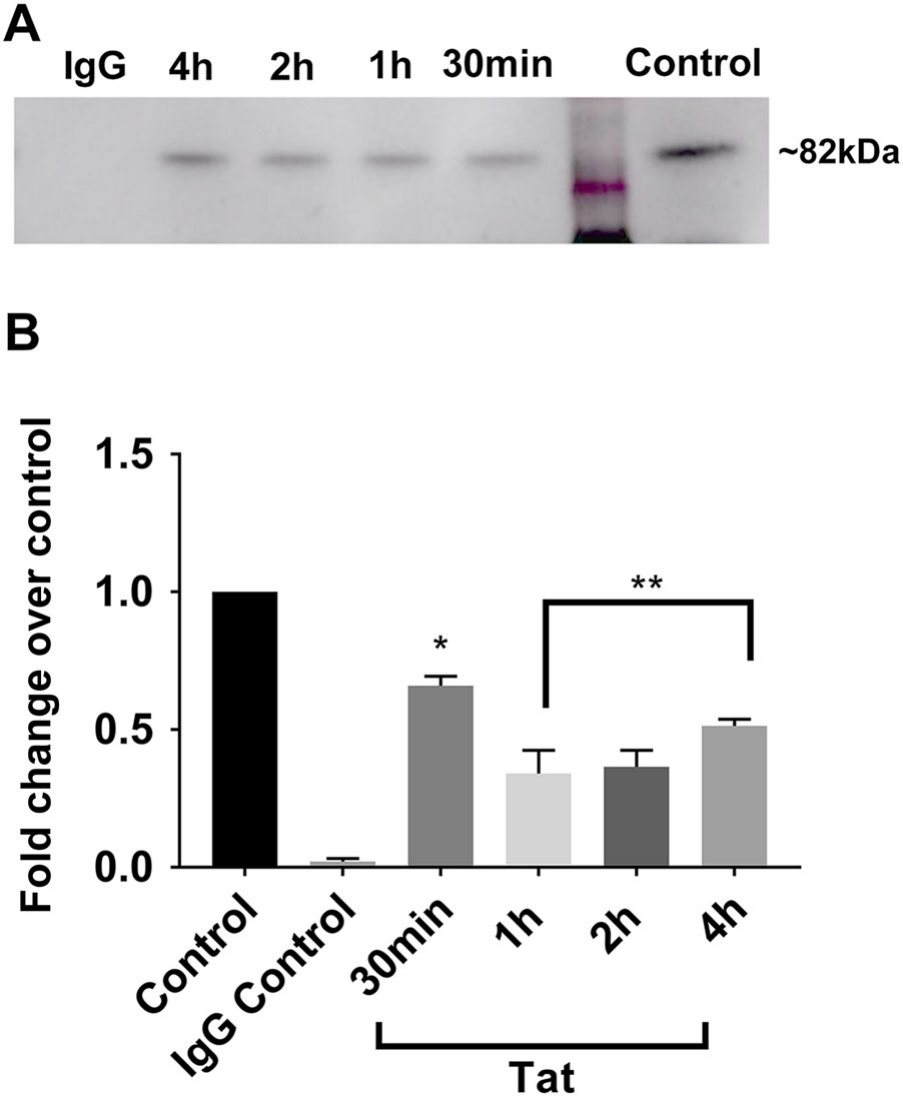
Tat decreases pDrp1 in cortical neurons in a time-dependent manner. Cortical neurons were exposed to Tat for the indicated times and then levels of pDrp1 were determined by western blot analysis in cell lysates following immunoprecipitation. **a** Representative western blot of lysates immunoprecipitated with anti-Drp1 antibody. The blot was analyzed with a pSer antibody. **b** Semi-quantification of pDRp1 immunoreactive band was done by densitometric analysis. Data, expressed as mean ± SEM, represent the average of three independent experiments. *p < 0.001, **p < 0.0001 vs control (ANOVA and Tukey’s test)

Increased mitochondria fragmentation and corresponding increase in Drp1 levels may prove to be related; nevertheless, Drp1 is a protein that can be found throughout the neuronal cytoplasm in its inactive form, whereas the active form of Drp1, which is most commonly dephosphorylated at Ser637^32^, is able to translocate to the mitochondrial membrane, where it initiates mitochondria fission. To examine whether Tat changes Drp1 phosphorylation (pDrp1), we performed an immunoprecipitation of lysates from Tat-exposed neurons with a Drp1 antibody, followed by western blot analysis with phosphoserine antibody (Fig. 6a). We found a decrease in pDrp1 levels in neurons exposed to Tat starting at 30 min and up to 4 h (Fig. 6b).

**Fig. 7:**
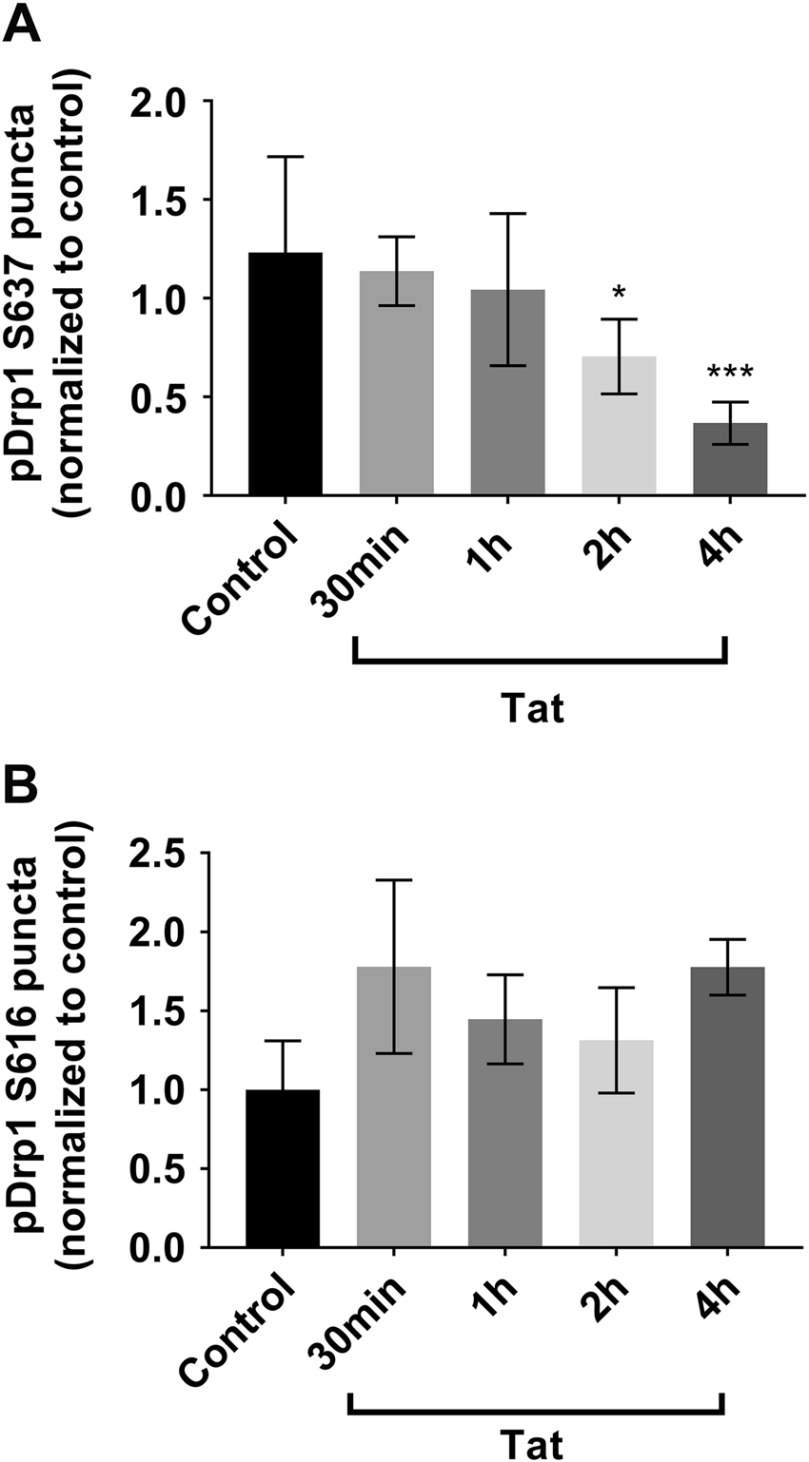
Tat decreases pDrp1 S637 puncta but not pDrp1 S616 puncta in a time-dependent manner. Cortical neurons were exposed to Tat for the indicated times, fixed, and then stained for pDrp1 S637 (**a**) or pDrp1 S616 (**b**). Quantification of pDrp1 S616 and pDrp1 S616 puncta was done as described in Materials and Methods. Data, expressed as mean ± SEM, are normalized to control and represent an average of three independent experiments (*n* = 10 neurons each experiment). *p < 0.05, ***p < 0.001 vs control (ANOVA and Tukey’s test)

Drp1 can be phosphorylated at several sites to regulate its activity^33^. Phosphorylation at S616 by Ca^2+^/calmodulin-dependent protein kinase Iα increases Drp1 activity, whereas phosphorylation at S637 or S656 by cyclic-AMP-dependent kinases decreases activity. To discern where changes in pDrp1 were taking place, we next performed immunocytochemistry on Tat-exposed neurons, examining pDrp1 S616 or S637. We observed a decrease in pDrp1 S637 puncta following Tat exposure (Fig. 7a), whereas pDrp1 S616 puncta remain unchanged (Fig. 7b), further supporting our observations of increased mitochondrial fragmentation and decreased levels of pDrp1. Taken altogether, these data indicate that the dephosphorylation of Drp1 at residue serine 637 may be contributing to the increased fragmentation of mitochondria following Tat exposure in neurons.

### Tat exposure increases calcineurin activity

**Fig. 8:**
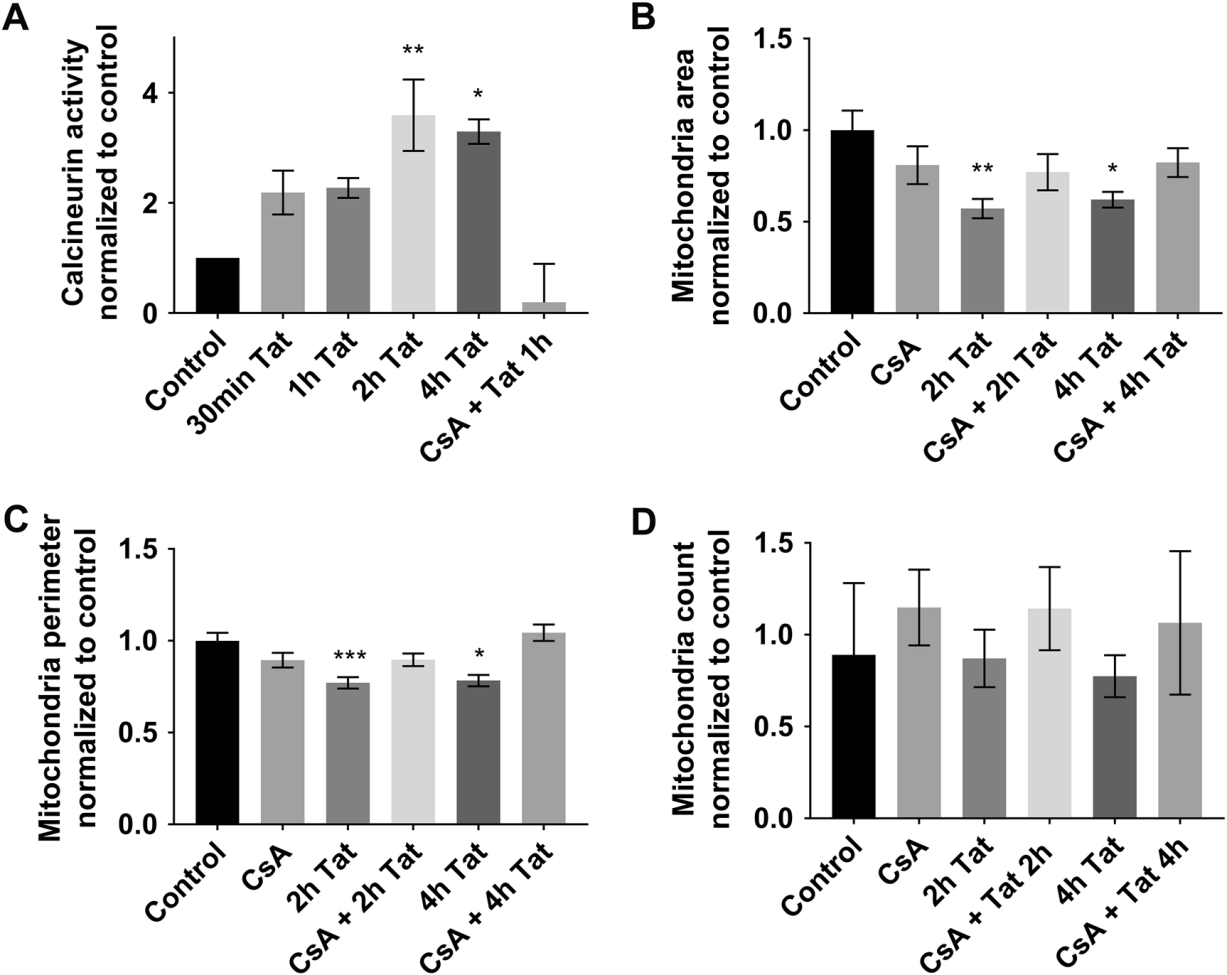
Calcineurin mediates the effect of Tat. **a** Cortical neurons were exposed to boiled Tat (control), Tat (100 nM), or to cyclosporin A (CsA, 10 μM) and Tat for the indicated times. Lysates were then collected and calcineurin activity was measured as described in Materials and Methods. Data, expressed as mean ± SEM, are normalized to control and represent an average of three independent experiments (*n* = 2 each time point each experiment). *p < 0.01, **p < 0.001 vs control (ANOVA and Tukey’s test). **b** Cortical neurons were exposed to Tat (100 nM), CsA (10 μM), or CsA + Tat for the specified time points. Cells were then fixed and stained for MAP2 and TOM20 to label neuronal microtubules and mitochondria, respectively, as described in Fig. 3. Quantification of mitochondrial area (**b**), perimeter (**c**), and number (**d**) was then done as described in Materials and Methods. Data are the mean ± SEM of 20 neurons, normalized to control. *p < 0.05, **p < 0.01, ***p < 0.001 vs control (ANOVA and Tukey’s test)

The GTPase activity of pDrp1 is regulated by the calcium-dependent serine/threonione phosphatase calcineurin^32^. The regulatory subunit of calcineurin, in turn, is activated by calmodulin^34^. Tat causes a fast and robust Ca^2+^ rise in neurons^31,35,36^, which can subsequently activate calmodulin^37^. Thus, it is plausible that neuronal exposure to Tat can cause the activation of Drp1 via a calcineurin-mediated mechanism. To determine whether Tat augments calcineurin activity, neurons were exposed to Tat for various time points from 30 min to 4 h and a calcineurin cellular activity assay was performed. Tat exposure caused a rise in calcineurin activity that was maintained for up to 4 h and was prevented by cyclosporin A (CsA; Fig. 8a), an inhibitor of calcineurin^38,39^.

To further establish a correlation between calcineurin, Drp1, and mitochondrial fission, neurons were exposed to a CsA 15 min prior to Tat, and TOM20 immunoreactivity was examined. We observed that in cultures pre-treated with CsA, which had no effect on mitochondrial numbers (Fig. 8d), Tat-induced mitochondrial fragmentation was mitigated (Fig. 8b, c). Together these results suggest that Tat-induced mitochondrial fragmentation is mediated in part by increased calcineurin activity in neurons.

## Discussion

The ability of Tat to induce neuronal damage and dysfunction in vitro and in vivo has been established^6^. Several mechanisms have been suggested to underlie the neurotoxic effect of Tat, including activation of *N*-methyl-d-aspartate (NMDA) receptors^31,36,40^, impairment of mitochondria physiology^13,41^, and DNA damage^14^. These mechanisms may ultimately participate in inducing neuronal apoptosis. In this study, we investigated whether the neurotoxic mechanism of Tat is mediated through the alterations of mitochondrial dynamics. Impaired mitochondrial dynamics, morphology, and distribution have been observed in post-mortem brains of patients suffering from HAND^15,16^, further identifying mitochondria as critical features for the pathology of HAND. Our data show that Tat elicits time-dependent changes in mitochondrial dynamics starting with their depolarization, followed by increased fission, leaving a mitochondrial population that is smaller in size than in controls. Mitochondria play a role in neuronal survival through a variety of mechanisms, including Ca^2+^ homeostasis^42^, as well as the control of ROS production^43^. Moreover, mitochondria regulate the synthesis of ATP, which is absolutely necessary for neuronal function^33,44,45^. In fact, disruption of energy because of mitochondrial dysfunction has been linked to numerous neurodegenerative diseases^20^. Thus, it is plausible to suggest that Tat-mediated mitochondrial damage is a key mechanism responsible for some of the neuronal impairments observed in HAND.

Our data show that Tat promotes a time-dependent depolarization of mitochondria. This effect is likely due to the rapid rise of intracellular Ca^2+^ caused by Tat, whether through NMDA receptor activation^36^, ryanodine receptors^12^, or l-type calcium channels^46^. Excessive mitochondrial Ca^2+^ loading causes a severe reduction in mitochondrial membrane potential and, in extreme cases, triggers apoptosis^47^. To protect against apoptosis, mitochondria alter their morphology through the dynamic processes of fusion and fission, allowing the mixing of matrix proteins, stabilization of membrane potential, and exchange of mitochondrial DNA (fusion) or the compartmentalization of damaged products (fission). In the current study, we present preliminary evidence that Tat enhances mitochondrial fission associated with increased activity of mitochondrial fission protein Drp1 and Ca^2+^-dependent phosphatase, calcineurin. Concurrent with these fluctuations in membrane potential is the significant fragmentation of neuronal mitochondria as well as the accumulation of mitochondria in the soma. These events occur days prior to Tat-induced apoptosis^14^, indicating the events are not synonymous with mitochondrial fragmentation observed in apoptosis. In fact, while induction of mitochondrial fission is necessary, it is not sufficient to stimulate neuronal apoptosis^48^.

In neurons, mitochondria must travel extreme distances and maintain energy homeostasis^19^. Neuronal mitochondria are distributed to regions of high metabolic demand, including synapses, nodes of Ranvier, and myelination/demyelination interfaces^49,50^. Moreover, synaptic plasticity and mitochondrial motility are highly positively correlated processes^51^. Thus, mitochondrial distribution is tied with the functional status of neurons. Mitochondria are abundant in synapses^52^, in keeping with their role of providing high energy required for synaptic transmission. Our data show that Tat alters mitochondrial distribution within neurons in a way that mitochondria in Tat-treated neurons are retrogradely transported from processes to the soma and accumulate around the nucleus. These mitochondria are smaller than those observed in control neurons. At the same time, we observed an increase in total levels of Drp1, consistent with an increase in fission. Increased Drp1 activity has been shown to contribute to neuronal injury and cell death^53,54^. Moreover, the co-exposure of neurons to Tat and Drp1 inhibitor mdivi-1 prevented Tat-induced mitochondrial fragmentation, further indicating that Drp1 activation is a necessary step in this neurotoxic process. These changes in mitochondrial morphology and subcellular localization can both contribute to neuronal destabilization, including the pruning of neurites and eventual cell death.

Translocation of Drp1 to mitochondria is regulated heavily by post-translational modifications, predominantly through phosphorylation and dephosphorylation. The phosphatase calcineurin plays a key role in these events. Indeed, calcineurin-dependent dephosphorylation of Drp1, at its conserved serine 637, regulates its translocation to mitochondria and increases GTPase activity as well^32^. In this study, we found that calcineurin activation corresponded with an increase in the active form of Drp1. Calcineurin has also recently been found to play a central role in protein misfolding in several neurodegenerative diseases^55^. Drp1 activity is also critical for neuronal survival. Excessive Drp1 activity is linked to neuronal death^56^, while downregulation of Drp1 leads to neuroprotection^48^. Our data show that Tat activates calcineurin and decreases the levels of pDrp1 S637. The immunosuppressant CsA, which has been used experimentally to prevent mitochondrial dysfunction^57^ and restore mitochondria-mediated synaptic plasticity,^42^ blocks the effects of Tat both on calcineurin activation as well as increased mitochondrial fragmentation, suggesting that Drp1 could play a role in Tat toxicity.

In conclusion, alterations in mitochondrial dynamics is believed to initiate neurodegeneration because they negatively influences energy distribution within synapses^58^. HIV infection of the central nervous system causes distinct mitochondrial alterations^15,16^. Experimentally, these effects occur even in the absence of the virus suggesting that Tat or another viral protein is sufficient to initiate an irreversible neurodegenerative process that may overlap with other endogenous neurotoxins or other pathophysiological insults. Our discovery provides new significant data for a better understanding of Tat-mediated neurotoxicity that will help in the design of adjunct therapies against HAND.

## Materials and methods

### Animals

All studies were carried out following the Guide for the Care and Use of Laboratory Animals as adopted and promulgated by the U.S. National Institutes of Health and approved by the Georgetown University and the University of California Animal Care and Use Committee.

### Reagents

Recombinant Tat was purchased from Immuno Diagnostics Inc. (Woburn, MA, USA). Mdivi-1 was purchased from Sigma-Aldrich (St Louis, MO, USA). Prior to each experiment, Mdivi-1 was dissolved into a solution by dimethyl sulfoxide (DMSO) and used at a concentration of 10 μM. CsA was purchased from Sigma-Aldrich and prepared prior to each experiment at a concentration of 10 μM in DMSO. FCCP was purchased from Tocris Bioscience (Minneapolis, MN, USA), TMRE from Abcam (Cambridge, MA, USA).

### Generation of inducible Tat-tg mice and doxycycline treatment

Inducible Tat-tg mouse colonies were obtained as previously described^29^. Briefly, a DNA fragment (2238 bp) containing the Teton-GFAP gene, along with downstream simian virus 40 splicing and polyadenylation sequences, was released by *Xho*I and *Pvu*II digestion of the pTeton-GFAP plasmid and purified by agarose gel electrophoresis and microinjected into fertilized eggs of F1 females obtained from mating between C3HeB and FeJ mice (The Jackson Laboratory, Bar Harbor, ME, USA). Founder transgenic animals were crossed with C57BL/6 mice to generate stable G-tg transgenic lines. Similarly, T-tg transgenic lines were obtained using a DNA fragment (1189 bp) released by *Xho*I and *Pvu*II digestion of the pTRE-Tat86 plasmid. Founder animals and progeny carrying the transgenes were identified by PCR analysis of genomic DNA, which was extracted from mouse tail clippings (0.5–1 cm long) using the Wizard genomic DNA isolation kit (Promega, Madison WI, USA). With this construct, mice express Tat upon doxycycline treatment (80 mg/kg). A total of 8 WT mice and 8 Tat-tg mice were used (7–8 months old).

### Electron microscopy

Vibratome sections from mouse brains were fixed, embedded, and sectioned with the ultramicrotome, as previously described^16^. To analyze the relative changes in average diameter of mitochondria, a total of 25 cells were analyzed per condition. Cells were randomly acquired from three grids. Grids were analyzed with a Zeiss OM 10 electron microscope as previously described^16^. Electron micrographs were obtained at a magnification of ×25 000. All the analyses of images were conducted on blind-coded samples. After the results were obtained, the code was broken, and data were analyzed with the StatView program (SAS Institute, Inc., Cary, NC, USA).

### Primary neuronal cultures

Cortical neuronal cultures were prepared from the cortex of embryonic (E17–18) Sprague–Dawley rats (Taconic, Derwood, MD, USA) following an established protocol^59^. Cells were seeded onto poly-l-lysine-precoated plates in neurobasal medium containing 2% B27 supplement, 25 nM glutamate, 0.5 mM glutamine, and 1 % antibiotic–antimycotic solution (Invitrogen, Carlsbad, CA, USA). Cultures were grown at 37 °C in 5% CO_2_/95% air for 7–8 days. Cultures contained ~10% of non-neuronal cells. Cells were exposed to control medium (heat-inactivated Tat in 0.1 % bovine serum albumin, BSA) or 100 nM Tat (in 0.1% BSA) for various time points. In experiments utilizing CsA and mdivi-1, each compound was applied to cultures as a 15 min pre-treatment.

### Stochastic optical reconstruction microscopy

STORM was performed using a Nikon A1 confocal microscope with CFI SR Apochromat TIRF ×100 oil objective and or Technology iXon3 897 EMCCD camera (Nikon Instruments Inc., Melville, NY, USA). Samples were treated according to the manufacturer’s protocol as previously described^15^. Rabbit anti-Tom20 (1:25; Santa Cruz Biotechnology Inc., Santa Cruz, CA, USA), mouse anti-Drp1 (1:1000; Abcam), and mouse anti-MAP2 (1:1000; Abcam) were used overnight to label mitochondria, Drp1, and neuronal cytoskeleton, respectively.

### Mitochondrial membrane potential assay

Neurons were grown on glass coverslips (DIV7) and were loaded with mitochondrial membrane potential-dependent dye, TMRE (Abcam) after exposure to Tat, FCCP (0.5 µM), and control media. After 30 min incubation at 37 °C, TMRE was washed from cells three times with 1× PBS warmed to 37 °C. Coverslips were transferred to glass slides for immediate imaging and subsequent fluorescence intensity measurement. Mean fluorescent intensity was measured using ImageJ (NIH, Bethesda, MD, USA).

### Western blots

Neuronal lysates were collected with 1×RIPA with protease and phosphatase inhibitors (Thermo Fisher Scientific, Waltham, MA, USA), sonicated, and spun at 10 000 r.p.m. at 4 °C for 10 min. Supernatant was collected and protein levels were measured (BCA Assay, Thermo Fisher Scientific). Samples were loaded into 4–12% Bis-Tris gel and run at 200 V, then transferred to a nitrocellulose membrane using iBlot 2 (Invitrogen). Membranes were blocked for 30 min (5% non-fat milk in PBS) at room temperature. Primary antibodies against Drp1 (Cell Signaling, Danver, MA, USA), TOM20 (Santa Cruz Biotechnology Inc.), and Mfn2 (Cell Signaling) were used at 1:1000 dilution. Anti-beta-actin (1:10 000, Sigma-Aldrich) was used as a loading control. Corresponding secondary antibodies conjugated to horseradish peroxidase were utilized and incubated for 2 h at room temperature or overnight at 4 °C. Immunoblots were developed using West Pico chemiluminescence (Thermo Fisher Scientific). Densitometry was performed using ImageJ (NIH).

### Immunoprecipitation

Magnetic beads (Invitrogen) were washed 2× in tris-buffered saline (TBS) with 0.2% Tween and then blocked for 1 h at room temperature in 1% BSA in TBS on a rotator. Supernatant was removed and beads were rinsed once with TBS. Following treatments, neuronal lysates were collected using 1× RIPA with protease and phosphatase inhibitors, and 1% Triton-X 100. Protein levels were measured using BCA Assay. Sample volumes with 200 μg protein were added to respective microcentrifuge tubes containing washed and blocked beads. Drp1 antibody was added to each sample tube at a dilution of 1:2000. A sample tube containing an IgG control antibody was also prepared. Double-distilled water was added to each sample to bring final volume to 500 µl. Labeled sample tubes were placed on a rotator at 4 °C overnight. The next morning, beads were isolated using a magnetic tube stand and the supernatant was removed. Beads were first washed 3× with ice-cold TBS-T, and then were resuspended in 50 µl of 1× loading buffer and boiled for 5 min. Again, a magnetic tube stand was used to separate the beads from the supernatant. Supernatants were removed and saved for immunoblots. Gels were loaded with 25 µl of each sample and run at 200 V using MOPS running buffer (Invitrogen). Proteins were transferred to nitrocellulose membranes using iBlot 2 transfer system. Protein levels were checked with Ponceau following transfer before probe. Blots were blocked with 5% BSA in TBS-T. Primary antibody (anti-pSer, Abcam) was prepared 1:1000 in 10% BSA in TBS-T and incubated on membranes for 1.5 h at room temperature. Secondary antibody was prepared 1:2000 in 5% BSA in TBS-T and incubated on membranes for 2 h at room temperature. Immunoblots were developed using West Pico chemiluminescence (Thermo Fisher Scientific). Densitometry was performed using ImageJ (NIH).

### Immunocytochemistry

Cells grown on coverslips were fixed in 4% paraformaldehyde and 4% sucrose for 15 min at room temperature. Cells were incubated 15 min in blocking solution (0.03% Triton-X, 5% non-fat milk or BSA in PBS) at room temperature. Primary antibodies were used at the following concentrations: TOM20 (1:1000, Santa Cruz); pDrp1 S637 (1:500, Cell Signaling); pDrp1 S616 (1:1000, Cell Signaling); Drp1 (1:1000, Abcam); and MAP2 (1:1000, Abcam). Incubation with primary antibodies was carried out overnight at 4 °C. Coverslips were washed with PBS and then incubated for 1 h at room temperature with the corresponding fluorescent secondary antibody (Invitrogen). Coverslips were then incubated with PBS containing 4′,6′-diamidino-2-phenylindole to visualize nuclei. Immunofluorescence was analyzed with a Leica SP8 confocal microscope system (Leica Microsystem, Buffalo Grove, IL, USA).

### pDrp1 puncta analysis

The ImageJ counting function was utilized to quantify pDrp1 puncta from obtained confocal images. In brief, images were converted to 8 bit and thresholded. A single neuron was outlined for analysis before the image was watershed so as to separate any adjacent but non-overlapping puncta, followed by particle analysis. All counts were normalized to the controls for each experiment and are presented as averages.

### Calcineurin activity assay

The calcineurin cellular activity assay kit (BML-AK816-0001, Enzo Life Sciences, NY, USA) was used to prepare neuronal lysates and test for calcineurin activity, following the manufacturer’s instructions. The absorbance values from the assay were converted into amount of PO_4_ released by calcineurin using the manufacturer’s instructions. All values were normalized to the values from control cells and plotted.

### Statistical analysis

The results of more than three independent experiments were compiled, and they were analyzed using Student’s *t*-test or one-way analysis of variance followed by Tukey’s post hoc test (GraphPad Prism 7, La Jolla, CA, USA). *P* values <0.05 were considered significant.
